# Alcohol use disorder and health-related quality of life in Korean night-shift workers: A cross-sectional study using the KNHANES 2007-2015 data

**DOI:** 10.1371/journal.pone.0214593

**Published:** 2019-04-01

**Authors:** Thu-Thi Pham, Boyoung Park

**Affiliations:** 1 National Cancer Center Graduate School of Cancer Science and Policy, Goyang, Republic of Korea; 2 Department of Medicine, Hanyang University College of Medicine, Seoul, Republic of Korea; Sefako Makgatho Health Sciences University, SOUTH AFRICA

## Abstract

This study aimed to evaluate the prevalence of night-shift work, and the association of night-shift work with alcohol use disorders(AUDs), as well as with health-related quality of life(HRQL), in Korean adult workers. A total of 26,895 adult workers aged 20–59 years were included in the analysis. AUDs were assessed using the Alcohol Use Disorders Identification Test(AUDIT), and HRQL was measured by the EuroQol-5D questionnaire with five main dimensions. We found an interaction effect between gender and working status on AUDs (p = 0.0065), suggesting that women are more fragile than men in terms of the effects of night work but not regarding HRQL (p = 0.1729). Female night workers had higher risk of AUDs than female day workers (odds ratio(OR): 2.23, 95% confidence interval (CI): 1.48–3.38) but this effect was not noted in male night workers (OR: 0.97, 95% CI: 0.69–1.37). Lower HRQL was found in depression dimension for night workers compared to day workers (OR: 1.37, 95% CI: 1.00–1.89), whereas day-night regular shift workers were protected from depression. Risk of AUDs and lower HRQL were identified in female night workers but not in male night workers. This association suggests that women are more fragile than men in terms of the effects of night work.

## Introduction

Night-shift work has negative effects on human biological adaption to the natural light and darkness cycles and cause health problems such as sleep disorders, gastrointestinal diseases, cardiovascular diseases, and cancers [1]. Night-shift work is defined as fulltime work that occurs between 24:00 and 05:00 [2]. In developed countries, about 20% of all employees perform work exceeding the regular working hours, and this proportion is likely to increase [1]. Therefore, the negative effects of night-shift work on health-related issues are being recognized now. Among the possible negative health effects of night-shift work, mental health has been less investigated compared with physical effects, and previous studies presented inconclusive results [3].

Alcohol use disorders (AUDs) include dependence, abuse, and harmful statements of alcohol use and increase in the proportion of health-care burden worldwide [4]. Night-shift workers who suffered poor sleep quality exhibit higher levels of alcohol consumption [5] and are at risk of AUDs [6]. However, previous studies regarding the association between night-shift work and AUDs showed inconsistent results and remain a source of controversy [6–8]. Some studies revealed no difference in alcohol consumption patterns between day and night workers [6, 7], while a study conducted on Norwegian nurses denoted a significantly positive association between hours worked per week and alcohol consumption [8]. A study conducted in an East Asia showed that service and sales workers had a greater risk of alcohol consumption than managers and professionals [9]. Korea is one of the countries in Asia with the highest prevalence of alcohol consumption, with its general population having a high-risk alcohol consumption rate of 15.1% [9].

Health-related quality of life (HRQL) refers to the subjective evaluation of the culture, society, and environment in which people are embedded [10]. One of the major factors affecting HRQL is the working status, such as occupation type, employment status, working hours, workplace condition, and shift work, including duration and frequency [11, 12]. Women involved in night works or rotating shifts showed lower HRQL compared with day workers [13]. However, a study conducted among Croatia hospital nurses denoted non-significantly lower HRQL in shift workers compared with non-shift workers [14].

Although the prevalence of night-shift work has increased and various health-related effects have been suggested, a limited relationship was established between night-shift work and its effects on mental health, especially in the Asian population [6, 9, 12]. Thus, this study aimed to examine the association between night-shift work and mental health focused on AUDs and HRQL applying nationally representative samples in an East-Asian country.

## Materials and methods

### Data source and study population

**Fig 1:**
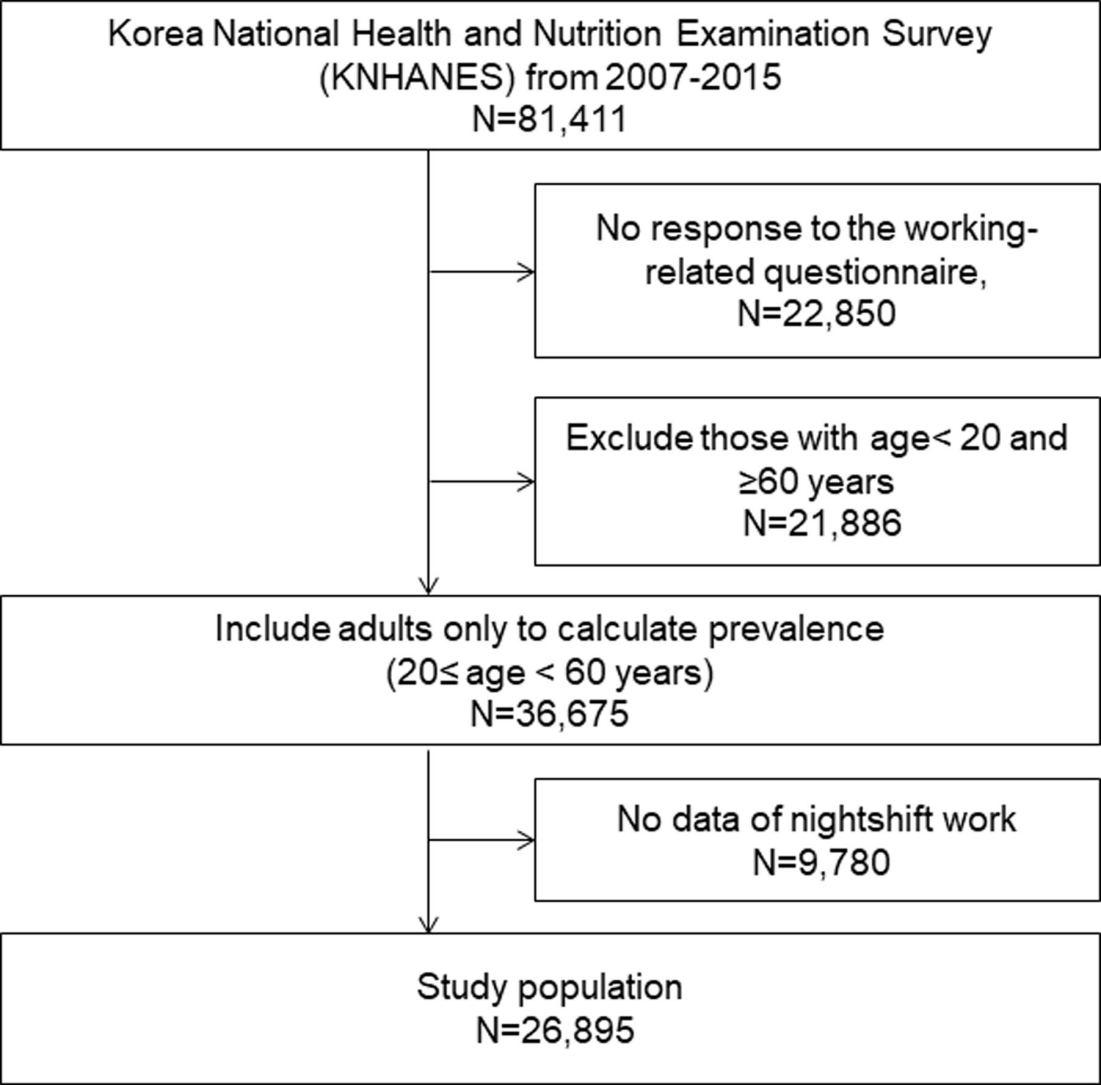
Flow chart of the selection process of study subjects from National Health and Nutrition Examination Survey IV, V, VI.

Data were obtained from the fourth (2007–2009), fifth (2010–2012), and sixth (2013–2015) Korea National Health and Nutrition Examination Survey (KNHANES) conducted by the Korea Centers for Disease Control and Prevention. The KNHANES is a nationally representative survey and used a multistage stratified cluster sampling method to the access data regarding the health and nutritional status of the Korean population since 1998. Health interview, health examination, and nutrition survey were the three main components of the KNHANES. The details of KNHANES were described elsewhere [15]. From 2007 to 2015, the number of study population was 81,411, with a 75% overall response rate. The study population was restricted to individuals aged 20–60 years, who responded to the questionnaire about working status, including 36,675 adults. Additionally, 9,780 individuals who had not had a job (N = 8,877), engaged in split work (N = 194), had a 24-hour rotating work (N = 154), with irregular working schedule (N = 288), or reported other working schedules (N = 267) during the last 1 year were excluded from the study. A total of 26,895 adults were included in the study (Fig 1). The KNHANES was approved by the Institutional Review Board of the Korea Centers for Disease Control and Prevention (IRB no. 2007-02CON-04-P, 2008-04EXP-01-C, 2009-01CON-03-2C, 2010-02CON-21-C, 2011-02CON-06-C, 2012-01EXP-01-2C, 2013-07CON-03-4C, 2013-12EXP-03-5C). All procedures were performed in accordance with the Declaration of Helsinki 7^th^ version and informed consent was obtained from all participants.

### Measurements

#### Working schedule and other covariates

In the KNHANES, after asking the participants if they have had a job during the last 1 year, the working schedule of the participants who ever had worked during the last 1 year was assessed. The major working schedules were classified as follows: mainly daytime work (6 am–6 pm), evening work (2 pm–midnight), night work (9 pm– 8 am the next day), day-night regular shift work, 24-hour rotating work, split shift, irregular shift work, or others. In this study, participants were considered as having day work if they had worked between 6 am and midnight in combination with daytime (6 am–6 pm) and evening work (2 pm–midnight), and night-shift if they had night work (9 pm–8 am next day) or day-night regular shift work. However, in the analysis, we divided night-shift work into two categories: night work and day-night regular shift work. Participants who reported other working schedules were excluded from the analysis due to the small number of participants. The baseline characteristics and lifestyles were recorded through interviewer-guided questionnaires, including age, gender, education, alcohol consumption, smoking status, average number of hours of sleep, stress, and physical activity.

#### Alcohol use disorders

The AUDs were assessed using the Alcohol Use Disorders Identification Test (AUDIT), with a scale from 0 to 40 [16]. Briefly, this test includes 10 questions related to hazardous alcohol use (frequency of drinking, typical volume, and heavy drinking), symptoms of alcohol dependence (ability to stop drinking, ability to control normal activities, and morning drinking), and harmful alcohol use (remorse after drinking, blackouts, alcohol-related injuries, and other concerns about drinking) as a simple method of screening for hazardous and harmful alcohol consumption in primary care setting [17]. We used the AUDIT cutoff value of ≥8 points to define AUDs. This was proven to give an appropriate level of sensitivity and specificity in unselected populations [16]. Additionally, based on the WHO guidelines, AUDIT scores were categorized into four zones with their corresponding interventions: zone I, 0–7 points: alcohol education; zone II, 8–15 points: simple advice; zone III, 16–19 points: counseling and continued monitoring; and zone IV, 20–40 points: referral to specialists for diagnostic evaluation and treatment [18].

#### Health-related quality of life

HRQL was measured by EuroQol-5D (EQ-5D), which was developed by the EuroQoL Group. EQ-5D was descriptively quantified by five dimensions, namely mobility, self-care, usual activities, pain/discomfort, and anxiety/depression. Each of the dimension was described as follows: 1  =  “no problem,” 2  =  “some problems,” and 3  =  “severe problems.” All of these response levels were converted into an EQ-5D index using the weight scoring system of the five dimensions ranging from 0 (worst) to 1 (best) [19]. The five dimensions were used as dichotomous variables by merging levels 2 and 3 into “some or extreme problem.”

### Statistical analysis

KNHANES data were analyzed, by applying sampling weights based on the inverse of selection probabilities, inverse of response rates, and other post-stratification factors including age, sex, and metropolitan area or province category [15]. The baseline characteristics were displayed by number, weighted percent, and p-values for chi-square tests for each working group.

The mean scores of AUDIT and EQ-5D were calculated and compared using weighted ANOVA with the Tukey’s post hoc test. The associations between night-shift work and AUDs as well as problem in HRQL 5 dimensions were evaluated using the complex samples logistic regression model adjusted for age, gender, education, smoking status, average number of hours of sleep, stress, and physical activity for the model of AUD. In the analysis between night-shift work and HRQL, we added alcohol drinking and removed stress as adjusted variables. In addition, we used the complex samples multinomial logistic regression model to analyze the association between night-shift work and the AUDIT of four categories. The interaction between night-shift work and gender was assessed by adding the interaction term into each model. The results of the analyses were expressed as odds ratios (ORs) and 95% confidence intervals (95% CIs). Data analysis was performed using SAS version 9.4 (SAS Institute, Cary, NC, USA).

## Results

The characteristics of the study population consisting of 13,571 (50.5%) male workers and 13,324 (49.5%) female workers are presented in Table 1. There were significant differences in gender, age, education, occupation, alcohol consumption, smoking status, and physical activity between working schedules (P-value <0.01).

**Table 1:** General characteristics of study population (n = 26,895).

Characteristic	Day work (N = 25,421)	Night work (N = 607)	Day-night regular shift work (N = 867)	P-value
	N ^a^ (%^b^)	N ^a^ (%^b^)	N ^a^ (%^b^)	
Gender				<0.0001
Male	12634 (57.85)	343 (65.65)	594 (73.91)	
Female	12787 (42.15)	264 (34.35)	273 (1.60)	
Age (years)				<0.0001
20–29	4194 (21.92)	184 (39.04)	167 (27.37)	
30–39	6681 (25.75)	115 (17.37)	285 (33.20)	
40–49	7488 (29.53)	157 (23.12)	241 (23.47)	
50–59	7058 (22.80)	151 (20.47)	174 (15.96)	
Education				<0.0001
≤Elementary school	2262 (7.30)	61 (8.49)	31 (2.92)	
Middle school	2480 (8.94)	69 (9.63)	59 (6.15)	
High school	9919 (41.50)	350 (62.27)	454 (54.38)	
≥ College	10752 (42.25)	127 (19.61)	323 (36.55)	
Occupation				<0.0001
Managerial and professional	10022 (39.05)	71 (11.34)	188 (20.73)	
Service and sales	4995 (19.28)	247 (41.83)	189 (20.47)	
Routine and manual	7547 (29.83)	200 (30.60)	425 (51.87)	
Others	2756 (11.84)	87 (16.23)	60 (6.93)	
Alcohol consumption				<0.0001
Ex-drinker/nondrinker	4427 (15.39)	80 (10.89)	95 (8.14)	
Current drinker	20950 (84.61)	525 (89.11)	771 (91.86)	
Smoking status				<0.0001
Ex-smoker/nonsmoker	16065 (66.72)	284 (46.25)	451 (57.78)	
Current smoker	6404 (33.28)	270 (53.75)	287 (42.22)	
Sleeping hour				
Less than 7 hours	18145 (71.46)	447 (71.24)	608 (70.24)	0.8487
7 hours or more	7156 (28.54)	156 (28.76)	257 (29.76)	
Stress				
No	17758 (70.13)	401 (67.96)	641 (73.32)	0.2198
Yes	7620 (29.87)	204 (32.04)	225 (26.68)	
Physical activity				
No	10366 (49.13)	209 (40.76)	315 (42.04)	0.0003
Yes	10386 (50.87)	290 (59.24)	390 (57.96)	

^a^ Net frequency of variable

^b^ Weighted percentage

**Fig 2:**
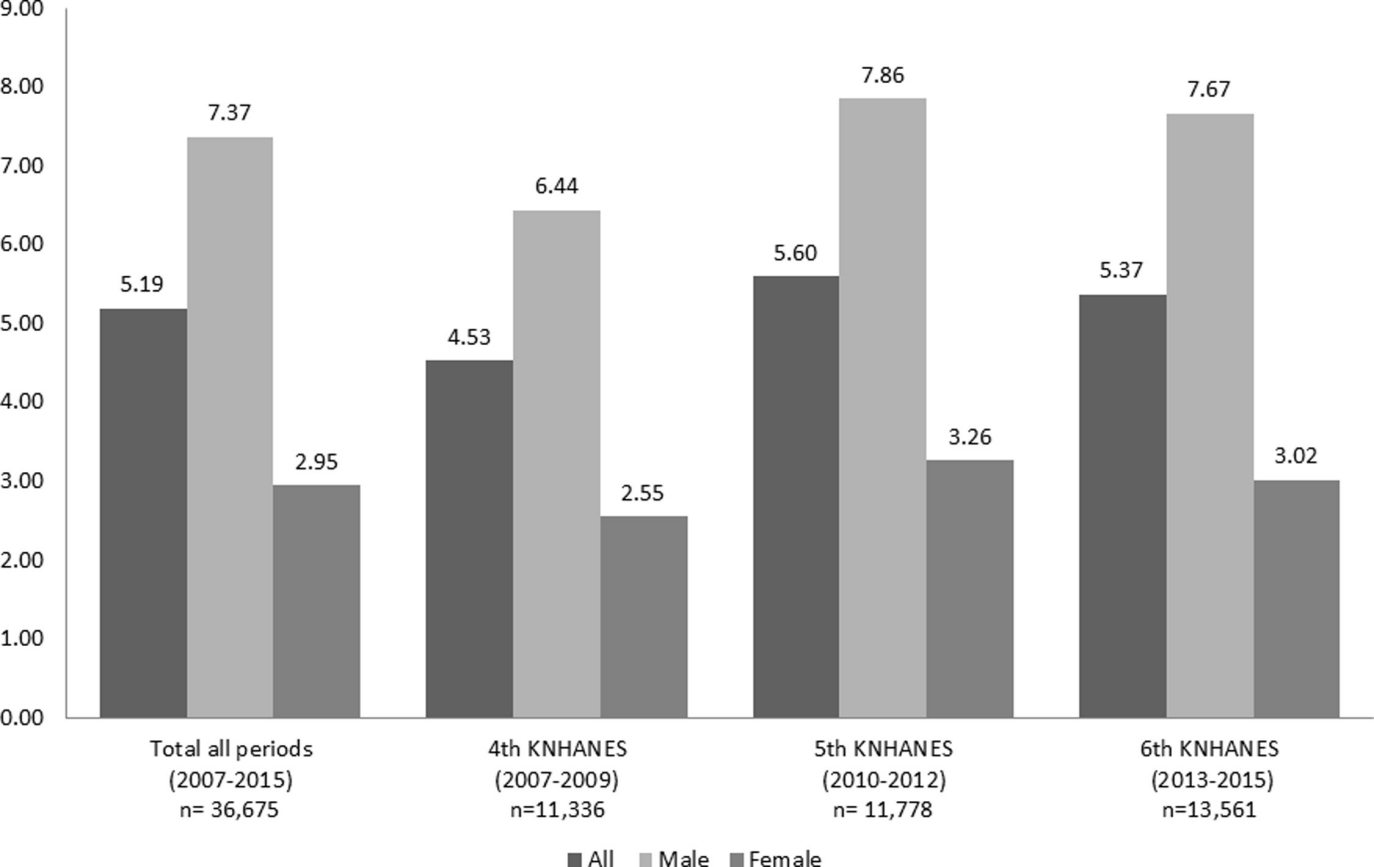
Prevalence (percentage) of last-one-year night-shift work according to the Korea National Health and Nutrition Examination Survey (KNHANES) through 3 periods included the fourth (2007–2009), the fifth (2010–2012) and, the sixth (2013–2015) in Korean adult workers 20≤ age < 60 years.

The mean age in the study population by working schedule were 41.4±10.6, 38.8 ±12.2, and 39.3±10.3 years for day work, night work, and day-night regular shift work, respectively. Approximately 2.26% of the study population (607 people) engaged in night work, and 3.22% (867 people) in day-night regular shift work. The weighted prevalence of night-shift work in the combined KNHANES IV–VI was 5.19%, with 4.53% in the KNHANES IV, 5.60% in the KNHANES V, and 5.37% in the KNHANES VI. In each survey period, the prevalence of night-shift work was higher among men than among women. Detailed proportions are provided in Fig 2.

The mean and standard deviation of AUDIT and EQ-5D scores among different working groups are presented in Table 2. The total population and women who engaged in night work showed significantly higher AUDIT scores (9.930 ± 0.444 and 8.299 ± 0.771) in the post hoc test, than those who engaged in day work and day-night regular shift work, but this association was not observed in men. For the EQ-5D index, day-night regular shift workers had a higher score than those in other working groups. Among women, night workers had significantly lower mean EQ-5D index than day-night regular shift workers (p = 0.0138 by Tukey’s post hoc test). The mean values of EQ-5D index in the three working groups were not significantly different among men (p = 0.1684). Furthermore, EQ-5D index were always lower for women than for men for each working group, with a statistical significance: the p-value for t-test of ED-5D index means by sex were <0.0001 in every working group.

**Table 2:** Means (standard deviation) of Alcohol Use Disorders Identification Test score and EuroQol-5D index in each working schedule.

	Alcohol Use Disorders Identification Test score ^c^			EuroQol-5D index ^c^		
	All	Male	Female	All	Male	Female
Day work	7.992 (0.066)	10.275 (0.092)	4.615 (0.076)	0.971 (0.001)	0.977 (0.001)	0.962 (0.001)
Night work	9.930 (0.444) ^a^	10.764 (0.538)	8.299 (0.771)^a^	0.965 (0.004)	0.976 (0.004)	0.945 (0.009)^b^
Day-night regular shift work	8.592 (0.326)	9.874 (0.385)	4.903 (0.519)	0.979 (0.002) ^a^	0.982 (0.003)	0.971 (0.004) ^b^
p-value (ANOVA test)	<0.0001	0.3967	<0.0001	0.0007	0.1684	0.0138

^a^ Superior to other groups based on the Tukey’s post hoc test

^b^ Significant difference with each other based on the Tukey’s post hoc test

^c^ For all analysis, the Alcohol Use Disorders Identification Test score and EuroQol-5D index score was significantly lower in female compared to male (P-value <0.0001)

Due to the mean differences in AUDIT score and EQ-5D index among different working schedules according to gender, we evaluated if there was an interaction between gender and working status on AUDIT score and EQ-5D index. The p-values of interaction were 0.0065 for AUDIT and 0.3943 for EQ5D; thus, we conducted both pooled and stratified analyses according to gender.

When an AUDIT cutoff value of ≥8 was applied to define AUDs, the weighted prevalence of AUD was 48.8% (63.3% in males and 24.8% in females) with 48.4% in day workers (63.5% in males and 24.3% in females), 59.4% in night workers (65.3% in males and 47.0% in females), and 50.2% in day-night regular shift workers (58.5% in males and 24.5% in females). The prevalence of those who experienced “some or extreme problem” in each dimension of the EQ-5D was 4.9% for mobility, 0.9% for self-care, 3.0% for usual activities, 16.4% for pain/discomfort, and 8.5% for anxiety/depression. The association between night-shift work and AUDs as well as HRQL is shown in Table 3. In women, the risks of AUDs were 2.23 times higher among night workers than among day workers. However, in men, no significant association was observed between night-shift work and AUDs. When categorizing AUDIT score into four zones, the risk of AUDs among women who engaged in night work substantially increased for zone II (OR: 1.88, 95% CI: 1.17–3.01), zone III (OR: 2.64, 95% CI: 1.22–5.73), and zone IV (OR: 4.58, 95% CI: 2.24–9.37) compared with zone I. However, no association was observed among men. The p-value for trend was <0.001 among female night workers compared with day workers (S1 Table). With regard to EQ-5D, only anxiety/depression dimension risk was observed to be significantly increased among night workers (OR: 1.37, 95% CI 1.00–1.89) and decreased among day-night regular shift workers (OR: 0.60, 95% CI: 0.40–0.91). A gender-stratified analysis showed that women engaged in night work had a higher anxiety/depression dimension risk than female day workers, but the significance disappeared when the OR was 1.45 (95% CI: 0.93–2.26). Otherwise, female day-night regular shift workers had a significantly lower anxiety/depression dimension risk than female day workers (OR: 0.46, 95% CI: 0.25–0.85). No significance was observed in the five dimensions of EQ-5D according to work type among men.

**Table 3:** The association between working schedules and alcohol use disorders, problems in 5 dimensions of EuroQol-5D.

Working times	Alcohol use disorders ^a^			EuroQol-5D		
		Mobility	Self-care	Usual activities	Pain/discomfort	Anxiety/depression
	OR (95% CI) ^b^	OR (95% CI) ^c^	OR (95% CI) ^c^	OR (95% CI) ^c^	OR (95% CI) ^c^	OR (95% CI) ^c^
All						
Day work	1	1	1	1	1	1
Night work	1.20 (0.89–1.62)	1.11 (0.70–1.75)	0.86 (0.38–1.94)	0.84 (0.47–1.51)	1.16 (0.88–1.54)	1.37 (1.00–1.89)
Day-night regular shift work	0.83 (0.66–1.06)	0.71 (0.42–1.21)	0.61 (0.25–1.52)	0.53 (0.24–1.17)	0.99 (0.74–1.31)	0.60 (0.40–0.91)
Male						
Day work	1	1	1	1	1	1
Night work	0.97 (0.69–1.37)	0.72 (0.33–1.56)	0.46 (0.14–1.49)	0.97 (0.45–2.07)	1.29 (0.89–1.87)	1.24 (0.73–2.10)
Day-night regular shift work	0.85 (0.65–1.10)	0.81 (0.42–1.56)	0.63 (0.23–1.76)	0.46 (0.13–1.61)	0.85 (0.61–1.19)	0.71 (0.41–1.24)
Female						
Day work	1	1	1	1	1	1
Night work	2.23 (1.48–3.38)	1.47 (0.80–2.68)	1.57 (0.56–4.40)	0.66 (0.27–1.63)	0.96 (0.64–1.44)	1.45 (0.93–2.26)
Day-night regular shift work	0.81 (0.50–1.33)	0.50 (0.24–1.06)	0.57 (0.08–4.23)	0.68 (0.30–1.51)	1.27 (0.82–1.97)	0.46 (0.25–0.85)

^a^ Alcohol Use Disorders Identification Test score <8 as reference group and ≥8 was defined as alcohol use disorders

^b^ Adjusted for (sex), age, education, smoking, sleeping hour, stress, and physical activity

^c^ Adjusted for (sex), age, education, alcohol drinking, smoking, sleeping hour, and physical activity.

## Discussion

The findings of this study showed a higher risk of AUDs and lower HRQL among night workers compared with day workers, especially in women. The risk of AUDs was 2.23 times higher among female night workers than among female day workers. In addition, an increased trend was observed in women engaged in night work with more severe level of AUDs. Female night-shift workers had a lower HRQL, measured by the EQ-5D index, than female day-night regular shift workers. However, these trends were not observed in men. Furthermore, women had significantly lower ED-5D index than men in every working group. These results suggest that women are more often affected by the effects of alcohol consumption and health-related problems even in the same night working status compared with men. To the best of our knowledge, this study is the first study to investigate the different associations between night-shift work and mental health in terms of AUD and HRQL according to gender.

The mechanism of the association between night work and AUDs could be explained by the intermediate effect of sleep disorder [20]. Shift work can cause sleep disorders, circadian rhythm disruptions, and disturbed sleep–wake pattern [21]. Insomnia patients are at a higher risk of AUDs and have higher alcohol consumption in order to improve their sleep [20]. Regarding sleep quality, men do not often suffer disrupted sleep likely because of less commitment to family responsibilities such as child care, but women experience more interrupted sleep due to family care both at night and beginning of the day, irrespective of their working schedule [22]. A gender gap in sleep quality caused by gender differences in working conditions and family responsibilities, such as household tasks and caregiving, has also been reported [23]. However, most of the previous studies showed similar or lower levels of alcohol consumption among night workers than among day workers [6–8] and it might be caused by that gender stratification was not performed. The individuals investigated in these studies were mostly employed as nurses, and involved a higher proportion of women; thus, stratified analysis could not be conducted [6, 8]. One study, in which 70% of the study population were men, did not conduct stratified analysis and also showed no association [7]. In our nationally representative study, we demonstrated the gender difference in the association between night work and AUDs for the first time. The heterogeneities of AUDs according to gender in the general population were denoted by many authors [4] [11]. Genevieve M et al. reported the four main components that influenced women’s drinking behavior: family drinking patterns, ethnic drinking patterns, gendered drinking norms, and occupational subculture [24]. Furthermore, some studies suggested that the effect of shift work differ according to gender due to the need to balance work and family roles, and women are more likely affected [25, 26]. Moreover, studies have reported that women experience more stress and gender discrimination but less reward than males in the same working conditions [27, 28] which may cause health-related consequences such as unhealthy use of alcohol. Other studies revealed that the increased vulnerability of women to the adverse effects of shift work would be caused by women’s physiological pattern, including more complex circadian and hormonal rhythms [29, 30].

With regard to HRQL, night workers are more likely to adopt to unhealthy lifestyles, which can lead to various adverse health-related outcomes including mental health problems [13], and decreasing the HRQL of night workers. The higher EQ-5D index and lower anxiety/depression dimension index in day-night regular shift workers could be due to their higher education level (Table 1). In addition, considering that the study population had a job during the last 1 year at the time of survey, other dimensions such as mobility, self-care, usual activities, or pain/discomfort might not decrease, but the anxiety/depression dimension was the most vulnerable among these dimensions. The gender differences in the association between night work and mental health, especially the higher risk trend among women, could be highlighted in this study despite the inconsistent results from several studies [3, 11, 29]. A cross-sectional study among Korean electronic workers denoted a statistically significant association between depression and shift workers in female but not in male [3]. However, in a large prospective cohort study, higher risks for developing depressed mood and depressive disorder in night-shift workers compared with day workers were not observed among either women or men [29]. In general population, similarly to our study’s findings, the effects of gender on the EQ-5D index have been reported that women had lower HRQL than men [31, 32]. Some studies revealed potential biases due to gender differences when using the quality of life instruments. Men and women’s response to the measurement of the HRQL or health utility were different even if they experienced a similar level of discomfort. In addition, some conditions that only women experience such as abnormal uterine bleeding could affect the differences or responses to the five dimensions in the EQ-5D and its related biases in various ways [33]. Women who engaged in night work experienced problems in their reproductive health that men never experienced [34]. Furthermore, the EQ-5D instrument includes questions about the participants’ experiences during the interview; thus, it may not reflect the HRQL that the participants experienced as a consequence of the night works during the last 1 year. In addition, the insignificant results in the stratification analysis despite of significances in the pooled analysis would be caused by the limited number of participants in each stratum.

Hence, we included participants aged 20–60 years, who were considered to be the main workforce of the population. The weighted prevalence of nightshift work was 5.19% during the 2007–2015 period, and men have a higher prevalence (7.37%) than women (2.95%). A survey conducted by the Korean Ministry of Employment and Labor (KMEL) reported that 11% of employees perform night work [35]. This discrepancy may be due to the fact that our interview questions were regarding the last 1-year night-shift exposure, whereas the questions in the KMEL survey asked about the participants’ lifetime exposure to night work. However, the exposure to the night work in Korean population was lower than that in Western countries [1].

The prevalence of AUDs was 48.8% with 48.4% in day workers, 59.4% in night workers, and 50.2% in day-night regular shift workers. Although direct comparisons were limited due to different measurement tools utilized, the prevalence of AUDs in Korean workers was higher than that of workers in other countries [36, 37]. However, the prevalence of the present study was comparable to other studies conducted in Korea [9, 38]. Considering the higher prevalence of alcohol consumption in Korea [9], it is likely that there could be a higher prevalence of AUDs in Korean workers compared with those of other countries.

This study had several strengths. We included a relatively large sample size, and the study population is representative of the general Korean population. Thus, the generalizability of the findings is guaranteed. However, this study also had some limitations. First, no causal relationship was established due to the cross-sectional design of the current study. In addition, because we did not have information about alcohol use and quality of life before the engagement in night work, reverse-causation such that females with AUDs or poor HRQL would be more engaged in night-shift jobs could be possible. Second, we could not assess the impact of frequency and intensity of night-shift work because the data are limited. Third, because we considered the work schedule during the last 1 year, the long-term effect of work schedule on AUDs or HRQL could not be assessed. Fourth, despite adjusted analysis was conducted to identify an independent association between night-shift work, AUDs, and HRQL, some possible variables affecting alcohol drinking patterns such as religion [39, 40] could not be considered because of lack of information. Fifth, despite the efforts to minimize the effect of possible covariates on outcomes such as adjustment and weighted analysis, the differences of selective factors between working schedules may have affected the results. Sixth, although AUDIT has been a widely used screening tool with satisfactory validity and reliability, it cannot replace the gold standard of clinical diagnosis [41, 42]. Thus, the findings of this study might not be concordant with those of clinically diagnosed AUDs.

In conclusion, the risk of AUDs was higher in women engaged in night work. The HRQL was lower in women engaged in night work than those engaged in day-night regular shift work. However, these associations were not observed in men. In addition, there was a gender difference in the risk of AUDs and HRQL in the same working status. Further studies should be conducted using a large prospective design and considering the frequency and intensity of night-shift work and risk of AUD and lower HRQL, to provide conclusive evidence for illumining these associations, in consideration with the different associations based on gender and the increased vulnerability in the female population.

## Supporting information

S1 Table Analysis the relationship between AUDIT score (4 zones) and nightshift working status using logistic regression and multinomial logistic regression with weighted adjustment. ^a^ AUDIT <8 as reference group. Adjusted for age, education, smoking, sleeping hour, stress, and physical activity.
(DOCX)
