# Supplementary material for: Alcohol use disorder and health-related quality of life in Korean night-shift workers: A cross-sectional study using the KNHANES 2007-2015 data
Source: PLoS One. 2019 Apr 1;14(4):e0214593. doi: 10.1371/journal.pone.0214593 (PMC6443159; doi:10.1371/journal.pone.0214593)
Supplement: S1 Table — a AUDIT <8 as reference group. Adjusted for age, education, smoking, sleeping hour, stress, and physical activity. (DOCX) [file pone.0214593.s001.docx]

**S1 Table.** Analysis the relationship between AUDIT score (4 zones) and nightshift working status using logistic regression and multinomial logistic regression with weighted adjustment

| Working times | AUDIT | | |  |
| --- | --- | --- | --- | --- |
|  | OR (95% CI) ^a^ | | | P-trend |
|  | Zone II (8-15) vs. Zone 1 | Zone II (8-15) vs. Zone 1 | Zone II (8-15) vs. Zone 1 |  |
| Male |  |  |  |  |
| Day work | 1 | 1 | 1 |  |
| Night work | 0.99 (0.68-1.44) | 0.99 (0.60-1.62) | 0.92 (0.57-1.50) | 0.912 |
| Day-night regular shift work | 0.89 (0.66-1.20) | 0.73 (0.49-1.09) | 0.81 (0.55-1.21) | 0.1649 |
| Female |  |  |  |  |
| Day work | 1 | 1 | 1 |  |
| Night work | 1.88 (1.17-3.01) | 2.64 (1.22-5.73) | 4.58 (2.24-9.37) | <0.0001 |
| Day-night regular shift work | 0.90 (0.53-1.53) | 0.50 (0.16-1.59) | 0.46 (0.11-1.90) | 0.5257 |

^a^ AUDIT <8 as reference group. Adjusted for age, education, smoking, sleeping hour, stress, and physical activity
